# The effects of surface structure mutations in *Arabidopsis thaliana* on the polarization of reflections from virus-infected leaves

**DOI:** 10.1371/journal.pone.0174014

**Published:** 2017-03-27

**Authors:** D. J. Maxwell, J. C. Partridge, N. W. Roberts, N. Boonham, G. D. Foster

**Affiliations:** 1 School of Biological Sciences, University of Bristol, Bristol, United Kingdom; 2 School of Animal Biology and Oceans Institute, University of Western Australia, Crawley, Western Australia, Australia; 3 The Food and Environment Research Agency, Sand Hutton, York, United Kingdom; The University of Tokyo, JAPAN

## Abstract

The way in which light is polarized when reflected from leaves can be affected by infection with plant viruses. This has the potential to influence viral transmission by insect vectors due to altered visual attractiveness of infected plants. The optical and topological properties of cuticular waxes and trichomes are important determinants of how light is polarized upon reflection. Changes in expression of genes involved in the formation of surface structures have also been reported following viral infection. This paper investigates the role of altered surface structures in virus-induced changes to polarization reflection from leaves. The percentage polarization of reflections from *Arabidopsis thaliana cer5*, *cer6* and *cer8* wax synthesis mutants, and the *gl1* leaf hair mutant, was compared to those from wild-type (WT) leaves. The *cer5* mutant leaves were less polarizing than WT on the adaxial and abaxial surfaces; *gl1* leaves were more polarizing than WT on the adaxial surfaces. The *cer6* and *cer8* mutations did not significantly affect polarization reflection. The impacts of *Turnip vein clearing virus* (TVCV) infection on the polarization of reflected light were significantly affected by *cer5* mutation, with the reflections from *cer5* mutants being higher than those from WT leaves, suggesting that changes in *CER5* expression following infection could influence the polarization of the reflections. There was, however, no significant effect of the *gl1* mutation on polarization following TVCV infection. The *cer5* and *gl1* mutations did not affect the changes in polarization following *Cucumber mosaic virus* (CMV) infection. The accumulation of TVCV and CMV did not differ significantly between mutant and WT leaves, suggesting that altered expression of surface structure genes does not significantly affect viral titres, raising the possibility that if such regulatory changes have any adaptive value it may possibly be through impacts on viral transmission.

## Introduction

It has been shown previously that virus infection can affect the percentage polarization of light reflected from leaves of *Nicotiana tabacum* and *Arabidopsis thaliana* [1], with possible implications for the transmission of viruses by insect vectors. In *N*. *tabacum*, the changes on the abaxial (lower) surfaces of leaves were associated with the viral transmission strategy; reflections from leaves infected with *Potato virus Y* (PVY) or *Cucumber mosaic virus* (CMV) (aphid vectored viruses) were less polarized in comparison to healthy leaves, whereas this effect was not observed with leaves infected with the non-insect vectored viruses *Tobacco mosaic virus* (TMV) or *Pepino mosaic virus* (PepMV) [1]. The polarization of the reflections was also affected in *A*. *thaliana*, although in this host there was little distinction between the impacts of CMV and the non-insect vectored virus *Turnip vein clearing virus* (TVCV) [1].

A key property that determines how reflected light is polarized is the structure of the reflecting surface itself: cuticular waxes and leaf hairs (trichomes) in the case of leaves [2–5]. Virus infection also affected the levels of expression of genes involved in the synthesis of epicuticular waxes [1]. Here we hypothesise that the altered expression of wax synthesis genes may contribute to differences between healthy and infected leaves in the polarization of the reflections. Trichomes are also known to influence the reflection of polarized light from leaves, with reflections for hairless (glabrous) leaves having a higher percentage of polarization compared to pubescent leaves [3]. However, previous work suggests that changes to polarization reflection during viral infection may not result from trichome phenotypes, as TVCV or CMV-infected *A*. *thaliana* leaves did not differ significantly in trichome densities from healthy leaves [1], although this may not be the case in other plant species.

In our study, polarization imaging was used to analyse the effects of *eceriferum* (*cer) 5*, *6* and *8* and *glabra1* (*gl1*) mutations on the percentage of linear polarization of light reflected from *A*. *thaliana* in blue and green wavebands. *CER5* is known to encode an ABC transporter protein which facilitates the movement of cuticular wax compounds across the cell membrane [6] resulting in the reduction of the total leaf wax load by 15% on *cer5* mutants [7]. The *cer6* mutant shows a 50% reduction in leaf wax load [8], with *CER6* being a condensing enzyme which catalyses the extension of fatty acid chains [9]. *CER8* catalyses the addition of coenzyme A to free fatty acids prior to their extension to very long chain fatty acids [10]; the total leaf wax load is unaffected in the *cer8* mutant, but alkanes are reduced whilst free fatty acids accumulate [10]. Finally, *GL1*, a Myb transcription factor, is required for trichome formation, with a total absence of trichomes on leaves of the *gl1* mutant [11]. Few studies report effects of viral infection on trichome formation, although it has been shown that in tomato plants infected with *Tomato yellow leaf curl virus* there are higher trichome densities on infected leaves than on uninfected leaves [12].

Altered expression of genes involved in the formation of leaf surface structures may affect host susceptibility or viral accumulation, as well as any effects on the leaf surface phenotype. For example, the expression of *PATHOGENESIS-RELATED PROTEIN 1* (*PR1*), involved in the systemic acquired resistance pathway, is greatly downregulated in the *cer6* mutant [13]. *RNA DEPENDENT RNA POLYMERASE* 1 (*RDR1*) reduces the spread of viruses in *N*. *tabacum* [14] and *A*. *thaliana* [15] due to the involvement of *RDR1* in the RNA silencing pathway, and is a suppressor of the *CER3* gene [16]. In *A*. *thaliana*, *MYB30*, a hypersensitive response regulator, is also a regulator of wax synthesis genes, with *CER2*, *CER3*, and *CER10* all being altered in transcript accumulation in the *myb30* mutant [17]. In this study, we compare the accumulation of TVCV and CMV in surface structure mutants and wild-type (WT) plants to establish whether altered expression of surface structure genes during viral infection could affect viral titres. To further investigate how viral infection may cause surface structure genes to change the polarization of the reflected light, the impact of TVCV and CMV infections on percentage polarization was compared between WT and mutant *A*. *thaliana*.

## Results

### Percentage polarization refection from healthy mutants and WT

Polarization imaging in blue and green wavebands was used to measure how much the light reflected from the adaxial and abaxial surfaces of rosette leaves from Arabidopsis L*er* WT and *cer5*, *cer6*, *cer8* and *gl1* mutants was polarized.

#### cer5

**Fig 1:**
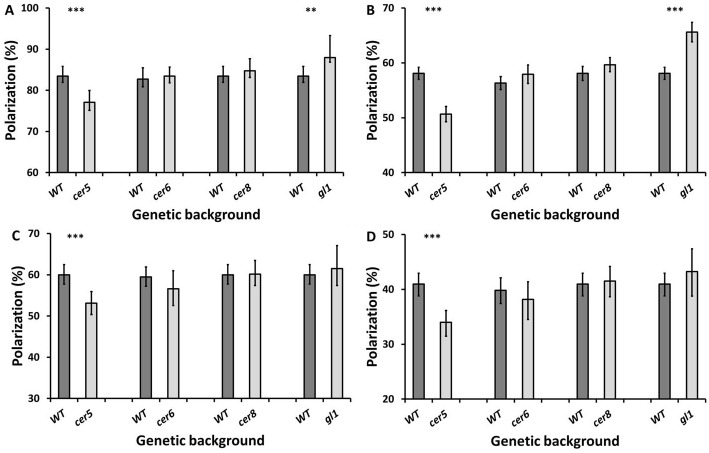
Average percentage polarization of light reflected from the adaxial (A,B) and abaxial (C,D) surfaces of *cer5*, *cer6*, *cer8* and *gl1* leaves, in comparison to the L*er* WT, in the blue (A,C) and green (B,D) channels. Error bars denote 95% confidence intervals of the means; asterisks indicate statistically significant differences between healthy and infected leaves (** *P*<0.01, *** *P*<0.001).

In the blue channel, the reflections from the adaxial surfaces of the *cer5* leaves were 6.34% less polarized than the WT (t-test, t = 4.12, df = 85, *P*<0.001) (Fig 1A) and 7.43% lower in the green channel (t-test, t = 3.692, df = 85, *P*<0.001) (Fig 1B). Similarly, reflections from the abaxial surfaces of *cer5* leaves were polarized 6.90% less in the blue channel (t-test, t = 3.938, df = 86, *P*<0.001) (Fig 1C) and 6.95% less in the green channel (t-test, t = 4.448, df = 86, *P*<0.001) (Fig 1D).

#### cer6

There was no significant different in the percentage polarization of the light reflected from the adaxial surfaces of *cer6* leaves in comparison to WT leaves, in either blue (t-test, t = -3.56, df = 66, *P* = 0.723) (Fig 1A) or green wavebands (Mann-Whitney test, z = -1.282, n = 68, *P* = 0.2) (Fig 1B). Similar results were obtained for light reflected from the abaxial surface, with no significant differences found in the blue channel (t-test, t = 1.178, df = 66, *P* = 0.243) (Fig 1C) or green channel (t-test, t = 0.889, df = 66, *P* = 0.377) (Fig 1D).

#### cer8

The *cer8* mutation also had no significant effect on percentage polarization of the reflection from the adaxial surfaces in blue (Mann-Whitney test, z = -1.699, n = 88, *P* = 0.089) (Fig 1A) or green (Mann-Whitney test, z = -0.872, df = 84, *P* = 0.383) channel light (Fig 1B). There was also no significant difference in the polarization of the light reflected from the abaxial surfaces in the blue channel (t-test, t = -0.171, df = 85, *P* = 0.864) (Fig 1C) or green channel (t-test, t = -0.323, df = 85, *P* = 0.748) (Fig 1D).

#### gl1

The adaxial surfaces of *gl1* leaves exhibited reflections that were 4.52% more polarized in the blue channel (t-test, t = -3.263, df = 84, *P* = 0.002) than WT leaves (Fig 1A) and 7.52% more polarized in the green channel (t-test, t = -3.337, df = 84, *P* = 0.001) (Fig 1B). In contrast, the percentage polarization of the reflections from the abaxial surfaces did not differ significantly between *gl1* and WT leaves, in both the blue (t-test, t = -0.823, df = 85, *P* = 0.413) (Fig 1C) and green wavebands (t-test, t = -0.952, df = 85, *P* = 0.344) (Fig 1D).

### Effects of *cer5* and *gl1* mutations on polarization reflection following TVCV or CMV infection

Given the significant influence of *cer5* and *gl1* mutations on polarization reflection, possible effects of the interaction between plant genotype and infection status on polarization reflection were analysed, to suggest whether altered regulation of these genes could potentially contribute to virus-induced alterations to polarization reflection.

#### TVCV

**Fig 2:**
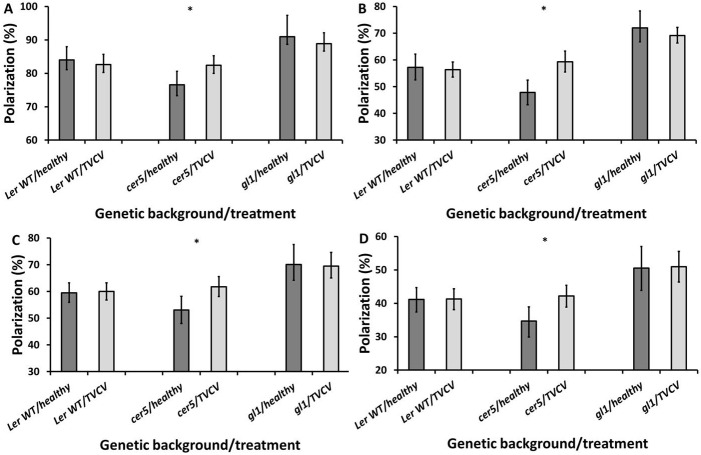
Average percentage polarization of light reflected from the adaxial (A,B) and abaxial (C,D) surfaces of healthy and TVCV-infected L*er* WT, *cer5* and *gl1* leaves, in the blue (A,C) and green (B,D) colour channels. Error bars denote 95% confidence intervals of the means; asterisks denote the cases where the effect of infection on the percentage polarization reflection from mutant leaves is significantly different to the effect on WT leaves (*P<0.05). Imaging was performed at 21 days post-inoculation, on systemically infected rosette leaves.

The effect of TVCV infection on how the adaxially reflected light was polarized differed between WT and *cer5* leaves (ANOVA, blue channel: F = 5.842, df = 1, *P* = 0.018; green channel: F = 10.144, df = 1, *P* = 0.002). The infection slightly reduced percentage polarization of the light in the WT, by 1.33% in the blue and 0.86% in the green channel. However, the polarization of the reflected light from the infected *cer5* leaves was higher than the healthy *cer5* leaves, by 5.89% and 11.51% in the blue and green channels respectively (Fig 2A and 2B).

A similar story is seen for the abaxial surfaces (ANOVA, blue channel: F = 4.794, df = 1, *P* = 0.032; green channel: F = 4.576, df = 1, *P* = 0.036). Increases of just 0.46% and 0.17%, in the blue and green channels respectively, were observed on the infected WT. However, the percentage polarization increased by 8.63% in the blue channel and 7.51% in the green channel from the infected *cer5* mutants compared with the healthy *cer5* leaves (Fig 2C and 2D).

The *gl1* mutation did not have any significant impact on changes in polarizing properties elicited by viral infection on adaxial leaf surfaces (ANOVA, blue channel: F = 0.048, df = 1, *P* = 0.827; green channel: F = 0.226, df = 1, *P* = 0.636) (Fig 2A and 2B) or the abaxial surfaces (ANOVA, blue channel: F = 0.055, df = 1, *P* = 0.815; green channel: F = 0.004, df = 1, *P* = 0.952) (Fig 2C and 2D).

#### CMV

**Fig 3:**
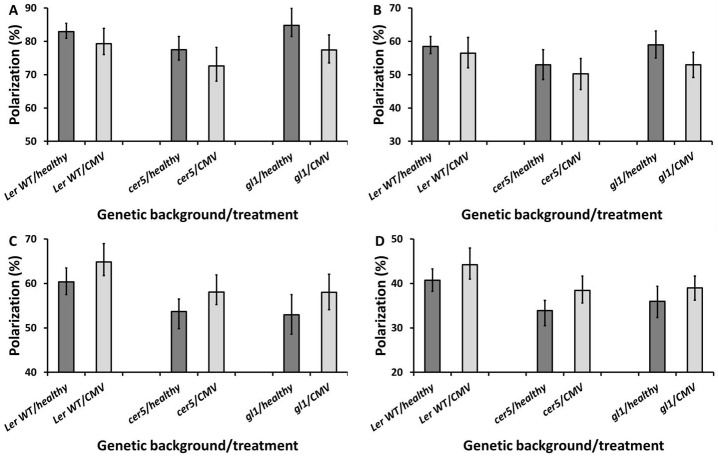
Average percentage polarization of light reflected from the adaxial (A,B) and abaxial (C,D) surfaces of healthy and CMV-infected L*er* WT, *cer5* and *gl1* leaves, in the blue (A,C) and green (B,D) colour channels. Error bars denote 95% confidence intervals of the means. Imaging was performed at 21 days post-inoculation, on systemically infected rosette leaves.

On the adaxial surfaces there was no significant effect of the *cer5* mutation on the percentage polarization of reflected light following CMV infection in the blue or green wavebands (ANOVA, blue channel: F = 0.11, df = 1. *P* = 0.741; green channel: F = 0.021, df = 1, *P* = 0.885) (Fig 3A and 3B). Likewise, on the abaxial leaf surfaces the effect of CMV infection on the WT was similar to on the *cer5* mutant, with no significant effect of the interaction of genetic background and treatment type on the percentage of polarization of the reflected light in blue or green channels (ANOVA, blue channel: F = 0.017, df = 1, *P* = 0.897; green channel: F = 0.354, df = 1, *P* = 0.553) (Fig 3C and 3D).

The *gl1* mutation had no significant impact on the polarization of light reflected from the adaxial surface following CMV infection (ANOVA, blue channel: F = 2.002, df = 1, *P* = 0.161; green channel: F = 1.02, df = 1, *P* = 0.315) (Fig 3A and 3B); or on reflections from the abaxial surface (ANOVA, blue channel: F<0.001, df = 1, *P* = 0.985; green channel: F = 0.031, df = 1, *P* = 0.861) (Fig 3C and 3D).

#### Viral accumulation in surface structure mutants

To establish whether mutations to surface structure genes may influence systemic viral accumulation, enzyme-linked immunosorbent assay (ELISA) was performed on the *cer5*, *cer6*, *cer8* and *gl1* genotypes following TVCV or CMV infection.

**Fig 4:**
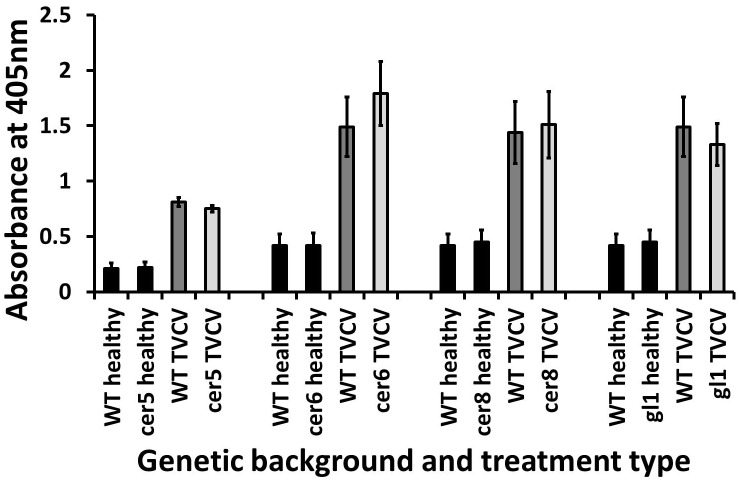
ELISA absorbance values from TVCV-infected rosette leaves of *cer5*, *cer6*, *cer8* and *gl1* mutants (light grey bars) in comparison to respective L*er* WT leaves (dark grey bars) at 14 days post-inoculation. Results from healthy control leaves are also shown (black bars). Error bars denote standard errors of the means.

Following TVCV infection there was no significant difference in viral accumulation between leaves of the WT and the *cer5* (t-test, t = 0.952, df = 31, *P* = 0.34), *cer6* (Mann-Whitney test, z = -1.18, n = 27, *P* = 0.254), *cer8* (Mann-Whitney test, z = -0.18, n = 29, *P* = 0.861) or *gl1* (Mann-Whitney test, z = -0.228, n = 30, *P* = 0.838) mutants (Fig 4).

**Fig 5:**
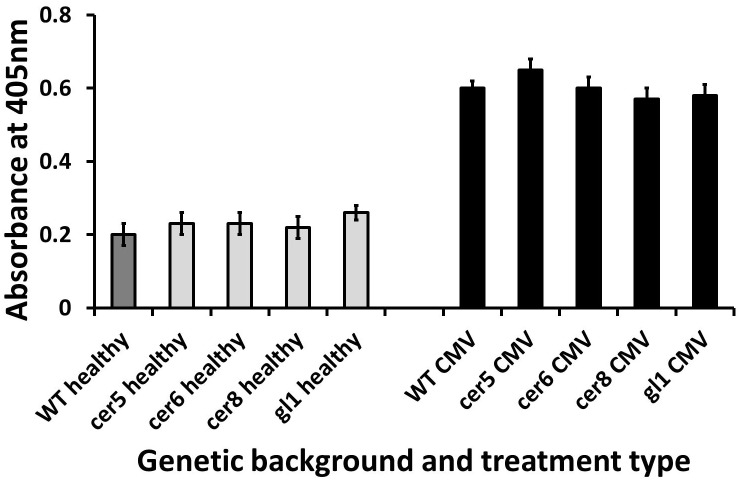
ELISA absorbance values from CMV-infected rosette leaves of *cer5*, *cer6*, *cer8* and *gl1* mutants (light grey bars) in comparison to L*er* WT leaves (dark grey bar) at 14 days post-inoculation. Results from healthy control leaves are also shown (black bars). Error bars denote standard errors of the means.

There was also no significant difference in CMV accumulation in *cer5* (t-test, t = -1.26, df = 27, *P* = 0.219), *cer6* (t-test, t = -0.044, df = 25, *P* = 0.965), *cer8* (t-test, t = 0.96, df = 27, *P* = 0.35) and *gl1* (t-test, t = 0.907, df = 26, *P* = 0.373) leaves in comparison to WT (Fig 5).

## Discussion

Polarization imaging comparing healthy WT and surface structure mutants suggests that mutations in genes which form leaf surface structures can affect the percentage polarization of the light reflected from leaves. Within the wax mutants, the percentage polarization of reflections from *cer5* leaves was lower than that reflected from WT leaves on both the adaxial and abaxial surfaces. However, none of the other mutants affected the percentage polarization of the light. A reduced accumulation of a particular type of wax is not likely to account for this difference, because no specific wax types are depleted to a greater extent on the *cer5* mutant than in the *cer6* or *cer8* mutants [7,8]. The total abundance of wax on *cer5* leaves is lowered by just 15% compared to the WT [7], whereas the depletion is 50% on the *cer6* mutant [8]. Possibly, the reduction in the polarization from the *cer5* mutant is a result of accumulated of waxes in epidermal cells, due to impeded transport of the constituents to the surface as a result of the *CER5* mutation (*CER5* encodes an ABC transport protein) [6]. This means the wax products accumulate in epidermal cell vacuoles [6] which could affect the turgor pressure of the cells, thereby influencing the smoothness of the leaf surface and hence percentage polarization of reflected light.

Previous work found that the aphid-transmitted viruses PVY and CMV caused increases in the abundance of *CER6* transcripts in the leaves of *N*. *tabacum*, and that these infections also led to significant decreases in the percentage of polarization of the light reflected from the abaxial surfaces of leaves [1]. However, the *cer6* mutation does not have any impact on the percentage polarization reflection in *A*. *thaliana*, suggesting that changes in the expression levels of *CER6* may not underlie these observed effects of PVY and CMV on polarization reflection, at least in this species.

With changes in chemical properties of the leaf cuticle there is also the potential to change the feeding behaviours of insect vectors as the epicuticular waxes have been shown to affect host discrimination by aphids [18]. If changes to the cuticle affect emissions of volatiles this may also affect the attractiveness of infected plants to vectors; it is well documented that volatiles have an influence on insect attraction to infected plants [19–23]. The links between waxes and pathogen defence systems discussed above [13–17] suggest that altered expression of wax synthesis genes could also have an adaptive value through effects on host susceptibility following infection.

It appears that the effects of virus infection on polarization of the reflections can also be affected by wax synthesis gene mutation, as the impact of TVCV infection on polarization reflection differed between the WT and *cer5* mutant (with little difference in percentage polarization being observed between healthy and infected WT leaves, but a notably increased percentage polarization in the case of infected *cer5* leaves compared with uninfected *cer5* leaves). This suggests that differential wax gene regulation may be involved in TVCV-induced alterations to leaf polarization reflection.

In contrast, CMV infection did not affect the percentage polarization significantly differently in the *cer5* mutants in comparison to WT. Differences in waxes therefore may not contribute significantly to CMV-induced alterations to polarization reflection.

It is unclear why there are differences in the effects of CMV and TVCV infection on the *cer5* mutant. However, it was previously found that TVCV infection downregulated *CER5* expression, whereas CMV did not induce this effect [1], so changes to waxes may play a role in bringing about the impacts on polarization reflection induced by infection with TVCV. The impacts of infection were not analysed in cer6 and cer8 mutants because leaves of these mutants showed no significant difference in percentage polarization reflection in comparison to WT. This does not eliminate the possibility that these mutations could influence the way infection impacts polarization reflection, although it does seem unlikely given the absence of *CER6* or *CER8* does not significantly affect polarization reflection from uninfected leaves.

On the adaxial surface, *gl1* leaves (which lack trichomes) were more polarizing than WT leaves. The significantly increased percentage polarization of light reflected from *gl1* leaves is consistent with previous studies suggesting that glabrous leaves are more polarizing than pubescent leaves [2,3]. However, this difference was only observed on the adaxial surfaces. This may be due to the cellular differences in the leaf interior between the two surfaces. The parenchyma cells of the abaxial surface scatter light reflected from the leaf interior more than the palisade cells within the adaxial surface scatter light [5] (hence the percentage polarization reflection tends to be higher in adaxially reflected light). This effect may reduce the relative influence of leaf hairs on polarization in light reflected from the abaxial leaf surface, leading to a lesser difference in percentage polarization between WT and *gl1* leaves on the abaxial surfaces in comparison to adaxial surfaces.

It does not appear that leaf hairs are important contributors to virus-induced changes to polarization reflection, as there was no difference in the impact of TVCV or CMV infection in the *gl1* mutant compared to WT. This supports previous work showing that TVCV or CMV infections did not affect trichome numbers on *A*. *thaliana* rosette leaves [1].

There are reported associations between genes involved in wax synthesis and plant defence pathways [13–17]. Therefore, any changes in expression levels of wax synthesis genes, and their possible phenotypic impacts on surface structures and the reflected polarization, could merely arise as a non-adaptive side effect of the interaction between host and pathogen at the level of defensive/counter-defensive mechanisms. This study suggests that the *cer5*, *cer6*, *cer8* and *gl1* mutants do not accumulate significantly different titres of CMV or TVCV compared to WT plants at two weeks following infection (although it remains possible that the rate of accumulation differs between the genotypes). It may therefore be the case that by affecting the regulation of leaf surface structure pathways, viruses gain some transmission enhancement; perhaps affecting the attractiveness or suitability of a leaf surface for insect vectors, or disrupting the surface in a way that facilitates mechanical transmission between plants for non-vectored viruses such as TVCV.

In summary, mutations of certain genes involved in wax biosynthesis and leaf hair formation can affect the percentage polarization of the light reflected from the leaves of *A*. *thaliana*. Furthermore, the effect of viral infection on polarization reflection also differed between a wax synthesis mutant and WT plants. The present results suggest that virus-induced wax gene expression changes may contribute to alterations to the leaf surface structure which could result in the differential polarized light reflection observed between healthy and infected leaves. The analysis comparing viral titres in wax mutants and WT leaves suggests that differential regulation of wax synthesis genes does not affect systemic viral accumulation; such changes could therefore have another adaptive value in plant-virus interactions. Given the prevalence of polarization sensitive visual systems in insects, vectored viruses could manipulate the attractiveness of virus-infected plants to their vectors through such changes, although in the present study there was no significant impact of *cer5* or *gl1* mutations on the effects of CMV infection on the percentage polarization of light reflected from leaves.

To further investigate these interactions between viruses, plants and insects it will be necessary to phenotypically analyse the waxy cuticle to understand whether and how infection alters the physical and chemical composition of the leaf surface; and to begin investigations into how visually guided behaviour of insect vectors of plant viruses is affect by the polarization of the scene.

## Methods

### Polarization imaging

Details of the polarization imaging process are given in [1]. In brief, multiple aligned images of leaves were acquired by rotating a linear polarizing filter held in front of the camera lens and data from the green and blue sensors of a Nikon DSLR camera were processed to provide information about the percentage polarization of all pixels in the image. Two independent biological replicates were performed for each virus; within each replicate 8–12 plants of each genotype were included within each treatment. For the comparison of healthy WT and healthy mutants, data obtained from the uninfected plants across these four replicates were pooled for analysis.

### Plant growth and inoculation

Seeds of *A*. *thaliana* were obtained from the Nottingham *Arabidopsis* Stock Centre (NASC IDs: *cer5*-N35, *cer6*-N6242, *cer8*-N40, *gl1*-N64), germinated at 20°C on Lehle medium (Lehle Seeds, TX, USA) in short day conditions (8:16 hours light:dark) and then grown for 14 days before being moved onto compost (Leavington F2 compost with added sand) for 14 days before viral inoculation.

Plants were mechanically inoculated with TVCV or CMV. Previously infected leaves were homogenised in deionised water and the sap was rubbed onto the adaxial leaf surface using carborundum powder as an abrasive. After two minutes this inoculum was washed off. Healthy controls were mock-inoculated with sterile deionised water only. Upper, expanding leaves in the rosette were selected for inoculation. The plants were then kept at 20°C, under short day conditions. Two independent biological replicates were performed for each virus; within each replicate 8–12 plants of each genotype were included within each treatment. For the comparison of healthy WT and healthy mutants, data obtained from the uninfected plants across these four replicates were pooled for analysis.

### ELISA

Between 14 and 18 plants of each mutant genotype were inoculated and compared to a similar number of infected WT plants. Systemically infected rosette leaves were selected for analysis.

Extraction, coating, substrate and wash buffers were obtained from Bioreba. Blocking solution comprised 5% (w/v) milk powder in PBS-tween (20mg PBS tablet (Sigma) dissolved in 200ml SDW, with 0.05% (v/v) tween-20).

For CMV assays a double antibody sandwich method was used. Antibodies were obtained from Bioreba and assays were performed according to the manufacturer’s protocols, with 50mg of leaf as the starting material.

For TVCV assays, a rabbit anti-TVCV coat protein primary antibody was kindly provided by Prof Ulrich Melcher at Oklahoma State University, and an alkaline phosphatase labelled anti-rabbit IgG secondary antibody from goat was purchased from Sigma. Indirect ELISA assays were performed according to the following protocol: 50mg leaf material was homogenised in 1ml coating buffer. 100μl was added to microtitre plate, incubated overnight at 4°C, and rinsed three times with wash buffer. 100μl blocking solution was added, incubated for two hours at room temperature, and rinsed three times with wash buffer. 100μl Primary antibody (diluted 1:10,000 in blocking solution) was added, incubated at room temperature for two hours and rinsed 3 times in wash buffer. 100μl secondary antibody was (diluted 1:30,000 in blocking solution) was added, incubated at room temperature for two hours and rinsed 3 times with wash buffer. 100μl para-Nitrophenylphosphate (pNPP) (dissolved in substrate buffer to 1mg/ml) was then added.

For both assays microtitre plates were incubated at room temperature for one hour after pNPP addition and read at 405nm on a VersaMax ELISA microplate reader (Molecular Devices).

### Statistical analysis

Analyses were carried out using SPSS statistics, version 19.0 (2010, IBM Corp.). In the polarization imaging analysis comparing uninfected WT and mutants, independent samples t-tests, or Mann- Whitney tests where data did not meet requirements for parametric tests (according to the Shapiro-Wilk test of normality) were used to test the for significance. Two-way ANOVA was used to test the significance of interactions between genotype and infection status on percentage polarization.

## Supporting information

S1 File (XLSX)

S2 File (XLSX)

S3 File (XLSX)

S4 File (XLSX)

S5 File (XLSX)
